# Statistical optimization for enhanced yields of probiotic *Bacillus coagulans* and its phage resistant mutants followed by kinetic modelling of the process

**DOI:** 10.1186/s40064-016-3325-8

**Published:** 2016-09-23

**Authors:** Kavita R. Pandey, Chetan Joshi, Babu V. Vakil

**Affiliations:** 1 GNIRD, G. N. Khalsa College, Matunga, Mumbai, 400019 India; 2 Department of Food Engineering and Technology, Institute of Chemical Technology, Matunga, Mumbai, 400019 India

**Keywords:** Probiotics, *Bacillus coagulans*, Random mutagenesis, Plackett–Burman methodology, Evolutionary operation (EVOP)

## Abstract

Probiotics are microorganisms which when administered in adequate amounts confer health benefits to the host. A leading pharmaceutical company producing *Bacillus coagulans* as a probiotic was facing the problem of recurring phage attacks. Two mutants viz. B. co PIII and B. co MIII that were isolated as phage resistant mutants after UV irradiation and MMS treatment of phage sensitive *B. coagulans* parental culture were characterized at functional and molecular level and were noted to have undergone interesting genetic changes. The non-specific genetic alterations induced by mutagenesis can also lead to alterations in cell performance. Hence, in the current study the parental strain and the two mutants were selected for shake flask optimization. Plackett–Burman design was used to select the significant culture variables affecting biomass production. Evolutionary operation method was applied for further optimization. The study showed wide variations in the nutritional requirements of phage resistant mutants, post exposure to mutagens. An increment of 150, 134 and 152 % was observed in the biomass productions of *B. coagulans* (parental type) and mutants B.co PIII and B.co MIII respectively, compared to the yield from one-factor-at-a-time technique. Using Logistic and modified Leudeking–Piret equations, biomass accumulation and substrate utilization efficiency of the bioprocess were determined. The experimental data was in agreement with the results predicted by statistical analysis and modelling. The developed model may be useful for controlling the growth and substrate consumption kinetics in large scale fermentation using *B. coagulans*.

## Background

Probiotics have been defined jointly by FAO and WHO as “Live microorganisms which when administered in adequate amounts confer a health benefit to the host” (FAO Joint 2007). Probiotics are mass produced by fermentation technology. Bacteriophages are the most notorious contaminants in dairy and probiotic industries, leading to cell death and hence, huge financial losses. One established and proven economic way to overcome the risk of phage attack is introduction of mutations to make the bacterial host genetically resistant to such attacks (Adsul et al. 2007). In the previous work, random mutagenesis was employed to obtain seven phage resistant mutants from the phage sensitive probiotic culture—*Bacillus coagulans* (Dubey and Vakil 2012). These mutants displayed variations in functional attributes in comparison to the parental culture (Pandey et al. 2015). Molecular characterization of the mutants revealed significant alterations in the genomic and proteomic profiles of mutants, compared to the parental profile. Partial 16SrRNA sequences of the mutants were provided with unique accession numbers by GenBank (Pandey et al. 2015). These alterations can be attributed to the use of mutagens which in turn might have introduced random mutations at multiple points throughout the DNA (Pandey et al. 2015). Owing to induced mutations the nutritional requirements of the mutants were altered as indicated by the “one-factor-at-a-time” (OFAT) technique. Hence, optimization of the medium composition and process conditions was considered necessary for the mutants.

Optimization of key process parameters, plays a vital role in process development, which in turn influences the cost of bioprocess (Bajaj et al. 2009). Culture medium optimization by traditional OFAT approach, requires considerable amount of time and labour (Cui et al. 2006). An alternative is to use statistical designs. Statistically based experimental designs provide a systematic and efficient plan for experimentation to achieve certain goals so that many factors and their interactions can be simultaneously studied. Therefore, in recent years number of statistical designs such as Plackett Burman (PB) design, factorial designing etc., have been used to rapidly identify the significant parameters influencing productivity. PB-design assists in screening of the important variables (five or more independent variables) affecting the desired product formation (Naveena et al. 2005). PB methodology allows evaluation of *n* variables in *n* + 1 experiments. Incorporation of dummy variables in an experiment makes it possible to estimate the variance of effects (Plackett and Burman 1946). Statistical optimization not only allows quick screening of large experimental domains, but also reveals the role of each component and their interactions with the other parameters (Kumar et al. 2011; Del Castillo 2007; Bae and Shoda 2005; Baskar and Renganathan 2009).

Evolutionary operation (EVOP) is a continuous process of optimization, which systematically determines the effects of two or three variables and their interactions at a time (Lynch 2003). The first step in implementing EVOP is identifying the pertinent process variables associated with an existing process (Bajaj et al. 2009). Then, a cycle of process runs are designed around the existing values of the process variables. Here, small changes are introduced deliberately in the process signal or process outputs to investigate their effects (Raissi and Farsani 2009). The changes made to variables from one cycle to the next are restricted and can only be made when the estimated improvements are greater than the estimated experimental error (Bajaj et al. 2009; Bankar and Singhal 2010).

The rationale design and optimization of the fermentation requires thorough understanding of production kinetics. A kinetic model can provide insight about the influence of operational parameters on cell growth, product formation and substrate utilization rate (Weiss and Ollis 1980). Thus it ensures the economic viability of a process. This information is important in order to identify the optimal operating conditions for maximal biomass accumulation (Shuler and Kargi 2002).

In the present study, Plackett–Burman design was adopted to identify the significant factors (glucose concentration, C/N ratio, agitation speed, temperature, pH, mineral concentration, size and age of inoculum), affecting biomass production. The most significant factors influencing the production were then optimized using EVOP. Further, simple logistic and Leudeking–Piret equations were developed for kinetic modelling of biomass production and substrate utilization, respectively.

## Methods

### Bacterial strain

Shake flask optimization was carried out for the parental strain and the previously obtained bacteriophage resistant mutants B.co-PIII (accession number-BankIt1761411 Bacillus KM652655) and B.co-MIII (accession number-BankIt1761402 Bacillus KM652654). These were phage resistant mutants obtained from a phage sensitive parental strain, using random mutagenesis technique (Dubey and Vakil 2010).

### Cultivation medium and culture conditions

The strains were cultured in glucose yeast extract broth (composition g/l—glucose: 10, yeast extract: 10, peptone: 10, sodium acetate: 10, magnesium sulphate: 0.1, potassium dihydrogen sulphate: 0.25, ferrous sulphate: 0.005, manganese sulphate: 0.005 and sodium chloride: 0.005; pH 6.8 ± 0.2) and incubated on orbital shaker at 37 °C. Optimization was carried out in 250 ml Erlenmeyer’s flasks containing 50 ml of glucose yeast extract broth. Each experimental set was carried out in triplicates.

### Statistical methodology

**Fig. 1:**
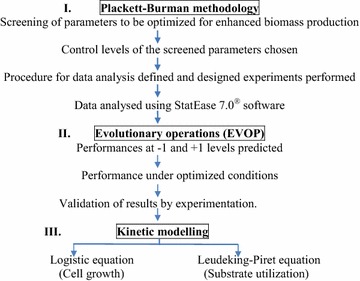
Schematic representation of the experimental plan (Walters et al. 1991)

In probiotic fermentations, the product of interest is biomass itself. Hence, shake flask optimization was carried out to achieve enhanced biomass production. Figure 1 depicts the scheme of work adopted for statistical optimization of shake flask cultivation conditions (for both, parent and two mutants), followed by kinetic modelling of the process.

### Screening of important physic–chemical parameters

To identify the variables which influence biomass production significantly, Plackett–Burman methodology was adopted using the tool design expert (Plackett and Burman 1946; Djekrif-Dakhmouche et al. 2006). On the basis of OFAT results (data not shown) parameters selected for the statistical optimization were glucose concentration, C/N ratio, agitation speed, temperature, pH, mineral concentration, size and age of inoculum. These variables have been enlisted in Table 1 (key) with low (−1) and high (+1) values. Dry cell weight (DCW) was analysed at intervals of 3 h during the 24 h incubation period. Standard deviation and error limits were calculated based on 95 % confidence limit. Data obtained from performed PB experiments were subjected to statistical evaluations like standard deviation, p value and regression analysis.

**Table 1:** Plackett–Burman design for shake flask optimization

Runs	Variables								Response (biomass—g/l)					
	A	B	C	D	E	F	G	H	*B. coagulans*		Mutant B. co PIII		Mutant B. co MIII	
									Act.	Pred.	Act.	Pred.	Act.	Pred.
1	−1	+1	+1	−1	+1	−1	+1	−1	3.58	3.585	3.89	4.293	2.89	2.843
2	−1	−1	+1	−1	+1	−1	−1	+1	3.57	3.862	3.03	3.535	2.21	2.243
3	+1	+1	−1	+1	+1	−1	−1	−1	2.95	3.198	3.89	4.183	2.52	2.400
4	+1	−1	−1	−1	+1	+1	+1	+1	2.75	2.720	2.61	3.425	2.07	2.297
5	+1	+1	−1	−1	−1	−1	−1	+1	4.52	4.542	4.12	4.183	2.75	2.837
6	−1	−1	−1	−1	−1	+1	−1	−1	2.37	2.13	2.41	2.303	2.37	2.157
7	+1	−1	+1	+1	+1	+1	−1	−1	5.35	5.043	5.1	4.657	3.41	3.317
8	+1	−1	+1	+1	−1	−1	+1	+1	4.8	4.877	4.91	4.657	3.23	3.257
9	+1	+1	+1	−1	−1	+1	+1	−1	6.12	6.110	5.89	5.415	4.48	4.353
10	−1	−1	−1	+1	−1	−1	+1	−1	2.67	2.882	2.82	2.303	1.64	1.660
11	−1	+1	+1	+1	−1	+1	−1	+1	4.23	4.173	4.03	4.293	3.57	3.777
12	−1	+1	−1	+1	+1	+1	+1	+1	2.98	2.772	3.61	3.062	2.32	2.320

The variable codes and their lower (−1) and higher (+1) values are as follows: A: glucose concentration—10 and 30 g/l, B: C/N ratio—20 and 40, C: agitation—150 and 250 rpm, D: temperature—30 and 40 °C, E: pH—5.5–7.5; F: mineral concentration—1× and 5×, G: inoculum size—5 and 10 % and age of seed—18 and 24 h, Act.: experimental data, Pred.: response predicted by model, responses are average of three values

### Optimization of screened parameters

EVOP methodology was used to optimize the selected parameters affecting biomass production (screened through PB experiments). The first process was run in five sets of conditions, in a random order and responses (yields—g/l) were recorded. In order to obtain higher yield, multiple sets of experiments were planned where, the best conditions of the previous set were used as the new search level of the next set. This was repeated till the optimum fermentation combinations were obtained yielding maximum biomass (g/l). Each set was run in triplicates. Analysis of variance (ANOVA) was used to estimate the significance of model coefficients. The experimental data were analysed with standard set of statistical formulae (Table 2) (Bankar and Singhal 2010).

**Table 2:** Calculation worksheet for analysis of effects, standard deviation and error limits for higher productivity

Effect of variables	Formulae to calculate effects of variables
Glucose	1/6 [X3 + X4 + X5 + X7 + X8 + X9] − [X1 + X2 + X6 + X10 + X11 + X12]
C/N	1/6 [X1 + X3 + X5 + X9 + X11 + X12] − [X2 + X4 + X6 + X7 + X8 + X10]
Agitation	1/6 [X1 + X2 + X7 + X8 + X9 + X11] − [X3 + X4 + X5 + X6 + X10 + X12]
Minerals conc.	1/6 [X4 + X6 + X7 + X9 + X11 + X12] − [X1 + X2 + X3 + X5 + X8 + X10]

X = response, C/N = carbon–nitrogen ratio

### Biomass and residual glucose determinations

Bacterial cell growth was determined by measuring the OD (A_540nm_). The biomass concentration was determined with a calibration curve made from the relationship between OD and DCW. The glucose concentration in the broth was determined by di-nitro salicylic acid (DNSA) assay, a spectrophotometric method (Sengupta et al. 2006).

### Kinetic modelling for the optimized process

Microbial fermentations which do not follow the classical kinetic model of substrate-limited biomass growth can be described using Logistic equation, which is an empirical function for sigmoid profile independent of substrate concentration (Rao et al. 2012). Modified Leudeking–Piret equation was applied to study the glucose utilization patterns of the probiotic strains (Weiss and Ollis 1980; Rajendran et al. 2007). Biomass production by *B. coagulans* (parental and mutant strains) and their substrate utilization patterns were evaluated in order to establish an unstructured mathematical model, which can be used to describe the corresponding kinetics.

## Results and discussion

### Screening of important physic–chemical components

PB-design of 12 experimental runs was constructed, where effect of each variable on biomass formation was determined by the equation given below:

$${\text{E}}_{{({\text{xi}})}} = 2\left( {\Sigma {\text{P}}_{{{\text{i}} + }} +\Sigma {\text{P}}_{{{\text{i}} - }} } \right)/{\text{N}}$$

where E(xi) = concentration effect of tested variable, P_i+_ and P_i−_ represent the response (yield) where variables were added at high and low levels respectively and N = no. of experiments.

The experimental data were analysed with standard set of statistical formulae enlisted in the Table 2.

Using PB methodology, three most significant variables influencing biomass production were identified for the three probiotic cultures viz. glucose concentration, C/N ratio and agitation speed. Combination of variables employed in experiment E_9_ in Table 1 (medium having 30 g/l of glucose, pH 5.5, 5× of minerals concentration and C/N ratio of 40:1, incubated at 30 °C, with agitation at 250 rpm when 18 h old seed was inoculated as 10 % (v/v) quantity) yielded the maximum biomass for all the three cultures. Maximal biomass production for the parental strain and mutants B. co PIII and B. co MIII were 6.12, 5.89 and 4.48 g/l respectively.

The p value indicates the significance of each coefficient, which indirectly reveals the interaction strength between each independent variable (Table 3). p values, <0.05 confirmed the credibility of models. The adequate precision measures signal to noise ratio (S/N) were 8.915, 10.037 and 8.779 for *B. coagulans*, mutants B.co PIII and B.co MIII respectively. All the values being >4 (desired value) ensured the reliability of the models (Bankar and Singhal 2010). The multiple regression analysis resulted in empirical models that relate the response measured to independent variables. Using PB-design variables having significant effect(s) on biomass accumulation were selected (Glucose concentration, C/N ratio and agitation speed), while the other statistically unimportant parameters like mineral concentration or pH, were eliminated. Variations observed in responses (yield—g/l) reflected the importance of statistical optimization to attain higher productivity. The regression equation obtained after ANOVA provided the levels of biomass produced as a function of initial values of A, B and C.

**Table 3:** Multiple regression analysis of the data obtained by PB-design

Cultures	SD (σ)	Adequate precision	p value	Model remark
Parent *B. coagulans*	0.63	8.915	<0.05	Significant
Mutant B.co PIII	0.54	10.037		
Mutant B.co MIII	0.43	8.779		

### Optimization of screened parameters

EVOP cycles were run till the most productive fermentation combinations were obtained. Process optimization for the mutants B. co PIII and MIII was achieved in three EVOP cycles each, whereas four EVOP cycles were run to achieve optimization for the parental type. Table 4 displays the combination of variables for EVOP cycle I. E_17_ yielded the maximum productivity for strain *B. coagulans* (7.1 g/l), while mutants B.co PIII and B.co MIII exhibited highest biomass accumulation in E_16_ (5.88 g/l) and E_15_ (4.79 g/l) respectively. It is noteworthy that yield of phage resistant mutants B.co PIII and B.co MIII were 82.82 and 67.46 % of the parental strain. C/N ratio suitable for maximum productivity was different for all the three strains (40, 35 and 30).

**Table 4:** Cycle-I of EVOP with five sets of conditions

	E13 (−1)	E14 (−2)	E15 (0)	E16 (+1)	E17 (+2)
Conditions					
Glucose (g/l)	5	6.25	7.50	8.75	10
C/N ratio	20	25	30	35	40
Agitation (rpm)	150	175	200	225	250
Response^a^ (biomass—g/l)					
Parent *B. coagulans*	4.80	5.72	6.10	6.97	7.10
Mutant B.co PIII	4.74	5.19	5.81	5.88	5.23
Mutant B.co MIII	3.79	4.02	4.79	4.38	4.25

^a^Responses are average of three values

Table 5(a) displays the EVOP cycles (II-IV) for optimization of shake flask conditions for the parental culture *B. coagulans*. As can be observed from the table, the interval difference for C/N was 5, and in the next three cycles they were 2, 1 and finally 0.5. Maximum productivity for the culture under study was 7.88 g/l and it was achieved in E_20_ with C/N ratio 42:1. The biomass accumulation of *B. coagulans* (parental type) after four EVOP cycles (7.88 g/l) was 28.75 % higher than the earlier yield of 6.12 g/l.

**Table 5:** EVOP designing for the three probiotic cultures and effects of error limits

Parameters	E18 (−1)	E19 (0)	E20 (+1)	E21 (−1)	E22 (0)	E23 (+1)	E24 (−1)	E25 (0)	E26 (+1)
(a) *B. coagulans* (parental strain)									
Glucose (g/l)	53	55.8	59	57	59	60	58	59	59.5
C/N ratio	38	40	42	41	42	43	41.5	42	42.5
Agitation (rpm)	200	225	250	240	250	250	245	250	250
Response^a^ (biomass—g/l)	7.2	7.38	7.88	7.2	7.6	7.42	7.3	7.5	7.2

Parameters	E18 (−1)	E19 (0)	E20 (+1)	E21 (−1)	E22 (0)	E23 (+1)
(b) Mutant B.co PIII						
Glucose (g/l)	38	44	50	47	50	53
C/N ratio	33	35	37	36	37	38
Agitation (rpm)	200	225	250	230	240	250
Response^a^ (biomass—g/l)	4.8	5.6	6.1	6.3	6.2	5.8

Parameters	E18 (−1)	E19 (0)	E20 (+1)	E21 (−1)	E22 (0)	E23 (+1)
(c) Mutant B.co MIII						
Glucose (g/l)	22.5	30	37.5	32.5	37.5	42.5
C/N ratio	28	30	32	31	32	33
Agitation (rpm)	175	200	225	220	230	240
Response^a^ (biomass—g/l)	4.82	5.2	5.56	6.1	6	5.8

Error limits	Formulae	*B. coagulans*	B.co PIII	B.co MIII
(d) Summary of effects of error limit values of EVOP cycles				
Averages	±1.414 × σ	0.577	0.409	0.363
Effects	±1.004 × σ	0.954	0.677	0.601
Change in mean	±0.891 × σ	0.814	0.578	0.513

σ—standard deviation

^a^Responses are average of three values

Optimization of shake flask conditions for mutants B.co PIII and B.co MIII were achieved in 3 EVOP cycles [Table 5(b, c)]. Combination in E_21_ worked out to be the best for both the mutants. Compared to responses generated earlier (5.89 g/l), yield after EVOP cycles (6.3 g/l) was marginally higher (1.07 times). Combination E_21_ yielded 6.1 g/l of biomass.

An estimated optimum response may not be optimum in reality. Errors in the estimates can lead to inadequacies of the model. Thus errors in estimations have to be taken into account. Banerjee and Bhattacharyya (1993) have put forward formulae to calculate the possible errors at 3 levels- error in average, effect and change in mean. The error limit values of average were lesser than the error limits for change in mean. Thus, the optimal conditions attained in the study are optimum in reality as well. Finally the optimized shake flask conditions for enhanced biomass production of the cultures under study are summarized in Table 6.

**Table 6:** Optimized shake flask conditions for maximum yields of probiotic strains

Variables	Optimized shake flask conditions for probiotic strains		
	*B. coagulans*	Mutant B. co PIII	Mutant B. co MIII
Glucose (g/l)	59	47	32.5
C/N ratio	42	36	31
Agitation (rpm)	225	250	220
Biomass^a^ (g/l)	7.88	6.3	6.1

^a^Responses are average of three values

### Kinetic modelling

The bacterial growth can be described by the following first order equation which states that the instantaneous rate of change in biomass is proportional to the quantity of biomass

1 $$dX/dt =\upmu\,X$$

where dx/dt is the growth rate (g/l/h), X is the specific growth rate (/h). The microbial growth is governed by a hyperbolic relationship and there is a limit to the maximum attainable cell mass concentration which is described by the logistic equation (Thirumavalavan et al. 2008).

The logistic equation has been used to evaluate µ.

2 $$\frac{dX}{dt} =\upmu\left[ {1 - \frac{X}{Xm}} \right]X$$

where µ denotes the specific growth rate (/h) and X_m_ is the maximum cell mass concentration (g/l). The maximum specific growth rate (µ_max_) for the three strains were around 0.4/h (Table 6). With increase in duration, biomass growth was accompanied with depletion of carbon source (glucose). Hence, substrate utilization kinetics, given by the modified Leudeking—Piret equation (below), was studied. Modified Leudeking–Piret equation considers substrate conversion to cell mass to product and substrate consumption for maintenance.

3 $$S = S0 - pX0\left[ {\frac{{e^{{\upmu mt}} }}{{\left( {1 - \frac{X0}{Xm}} \right)\left( {1 - e^{{\upmu mt}} } \right)}} - 1} \right] - qXm\upmu m\,\ln \,1 - \frac{X0}{Xm}\left( {1 - e^{{\upmu mt}} } \right)$$

where So and S are the substrate concentrations at time 0 and t, respectively. The values of the biokinetic parameters for biomass formation and substrate (glucose) utilization by *B. coagulans* have been enlisted in Table 7. The values of α (growth associated constant) for *B. coagulans* and its mutants was around 6.5. Low β values indicate the negligible growth occurred in the stationary phase. Regression analysis was performed to assess the credibility of each model. R^2^ values for logistic as well as Leudeking–Piret equations for the three probiotic strains were above 91 %, indicating the reliability of the kinetic model.

**Table 7:** Maximum growth rates obtained on kinetic modelling of the three cultures

S. no.	Strain	Actual µ_max_	Predicted µ_max_	X_m_
1	Parental *B. coagulans*	0.408	0.40	0.41
2	Mutant MIII	0.389	0.399	0.4
3	Mutant PIII	0.396	0.40	0.4

**Fig. 2:**
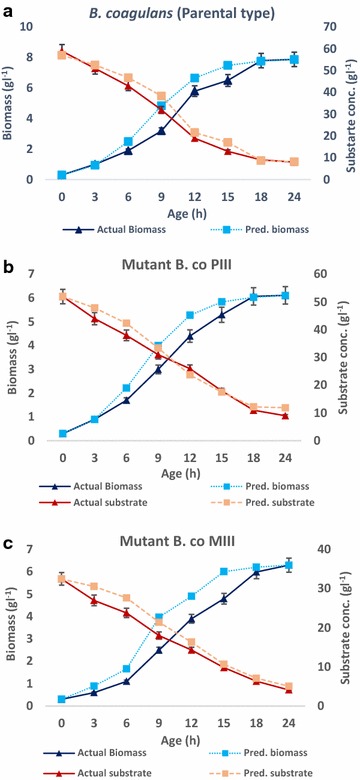
Plots of biomass produced (experimentally) by the 3 probiotic cultures—*B. coagulans* (parent) (**a**) and mutants B.co PIII (**b**) and B.co MIII (**c**), against the values predicted by model (experiments were run in triplicates)

Substrate concentration gradually decreased with time correlated with enhanced biomass accumulation. Maximum glucose supplement was used for cell multiplication and energy generation. Figure 2a–c shows comparison of experimental and model predicted values for shake flask level biomass growth and substrate utilization, respectively. The R values for biomass accumulation by the probiotic strains were 0.935, 0.90 and 0.968 for *B. coagulans* parental type and mutants B.co PIII and B. co MIII, while substrate utilization trends displayed R values of 0.963, 0.905 and 0.952. Close resemblance of experimental and model values had validated the proposed models.

The experimental results were very much in accordance with the results predicted by models hence best for the optimized process (Fig. 2a–c). Maximum biomass obtained for the probiotic strains *B. coagulans* and mutants PIII and MIII were 7.88, 6.3 and 6.1 g/l respectively.

As may be noted from the data presented earlier, biomass produced by mutants B.co PIII (6.3 g/l) and B.co MIII (6.1 g/l) were almost equal, but at two different C/N ratios of 36 and 31 respectively. Mutant B.co MIII appears to be best culture for commercialization as it is not only phage resistant but also yields 77.4 % biomass utilizing only 55.40 % amount of glucose as compared to the parental culture. Further work on optimization for this mutant can result in production of biomass equivalent to or even more than the phage sensitive parental culture.

Mandenius and Brundin (2008) have reviewed several examples of enhancing productivity using statistical designing. Several reports have confirmed the effectiveness of statistical optimization in the production enhancement (Brinques et al. 2010; Kumar et al. 2012; Annapurna 2009). Statistical modelling of *B. longum* resulted in process optimization such that the glucose requirement decreased by 50 % and yield increased by 160 % (Meena et al. 2011). Productivity of jiean peptide—JAA was enhanced by 41 % in *B. subtilis* ZK8 cells by statistical optimization (Zhang et al. 2010). Fermentation conditions for production of 2, 3 butanediol by *K. pneumoniae* were optimized using statistical approaches (Song et al. 2012).

The maximum specific growth rate obtained for the three probiotic strains was around 0.4/h. The results are in good agreement with µ_max_ values reported for *Bacillus subtilis* (0.49/h) and *B. thuringiensis* (0.38/h) (Rivera 1999; Chen et al. 2004). However, there are reports of some Bacillus strains displaying a wider range of µ_max_ values (*B. thuringiensis* (0.54/h) and *B. thuringiensis* var kurstaki 1.1/h) (Rao et al. 2012; Anderson 1990; Avignone-Rossa and Mignone 1995; Monroy and De La Torre 1996).

Shake flasks are always used as a rapid and primary system for screening and optimization of a microbial process before moving the operations to fermentors. A shake flask and fermentor are quite different systems in the context of geometry, mixing and oxygen availability and lack the possibility to monitor the fermentation during the experiment. This necessitates a further statistical optimization of bioprocess for *B. coagulans* fermentation at lab scale.

## Conclusions

Shake flask studies as anticipated showed wide variations in the process conditions required for maximum growth of the three probiotic cultures. Using Plakett–Burman methodology the three most significant variables affecting biomass production were identified viz glucose concentration, C/N ratio and agitation speed. Parameters like mineral concentration and pH had negligible effects. EVOP was applied to the data obtained by PB-design to predict the optimum conditions leading to maximal biomass formation. Application of statistical method—EVOP resulted in significant enhancement of 150, 134 and 152 % in the biomass production by *B. coagulans* parental type, mutants B.co PIII and B.co MIII respectively compared to the yield from OFAT approach.

Thus, it can be inferred that use of random mutagenesis not only resulted in isolation of phage resistant mutants but it also had its effect on the nutritional requirements and growth pattern of the mutants. Growth pattern followed the logistic model, while substrate utilization trend followed the modified Leudeking–Piret equation. The experimental data was in agreement with the results predicted by statistical analysis and modelling ensuring the credibility of the model. The developed model may be useful for controlling the growth and substrate consumption kinetics in large scale fermentation using *B. coagulans*.

## References

[CR1] Adsul MG, Bastawde KB, Varma AJ, Gokhale DV (2007). Strain improvement of *Penicillium janthinellum* NCIM 1171 for increased cellulase production. Bioresour Technol.

[CR2] Anderson T (1990) Effects of carbon: nitrogen ratio and oxygen on the growth kinetics of *Bacillus thuringiensis* and yield of bioinsecticidal crystal protein. M. Sc. thesis, University of Western Ontario

[CR3] Annapurna A (2009). Cardio protective actions of two bioflavonoids, quercetin and rutin, in experimental myocardial infarction in both normal and streptozotocin-induced type I diabetic rats. J Pharm Pharmacol.

[CR4] Avignone-Rossa C, Mignone CF (1995). *Bacillus thuringiensis* growth and toxicity. Mol Biotechnol.

[CR5] Bae S, Shoda M (2005). Statistical optimization of culture conditions for bacterial cellulose production using Box–Behnken design. Biotechnol Bioeng.

[CR6] Bajaj V, Mak-Jurkauskas M, Belenky M, Herzfeld J, Griffin R (2009). Functional and shunt states of bacteriorhodopsin resolved by 250 GHz dynamic nuclear polarization–enhanced solid-state NMR. Proc Natl Acad Sci.

[CR7] Banerjee R, Bhattacharyya B (1993). Evolutionary operation (EVOP) to optimize three-dimensional biological experiments. Biotechnol Bioeng.

[CR8] Bankar S, Singhal R (2010). Optimization of poly-ε-lysine production by *Streptomyces noursei* NRRL 5126. Bioresour Technol.

[CR9] Baskar G, Renganathan S (2009). Statistical screening of process variables for the production of L-asparaginase from cornflour by *Aspergillus terreus* MTCC 1782 in submerged fermentation. Indian J Sci Technol.

[CR10] Brinques GB, do Carmo Peralba M, Ayub MAZ (2010). Optimization of probiotic and lactic acid production by *Lactobacillus plantarum* in submerged bioreactor systems. J Ind Microbiol Biotechnol.

[CR11] *B. coagulans* bacteriophage resistant mutant GNKC/PBc/MMSm3: BankIt1761402: Bacillus KM652654. http://www.ncbi.nlm.nih.gov/nuccore/768030656

[CR12] *B. coagulans* bacteriophage resistant mutant GNKC/PBc/UV m3: BankIt1761411 Bacillus KM652655. http://www.ncbi.nlm.nih.gov/nuccore/768030637

[CR13] Chen QH, He GQ, Schwarz P (2004). Studies on cultivation kinetics for elastase production by *Bacillus* sp. EL31410. J Agric Food Chem.

[CR14] Cui F, Li Y, Xu Z, Xu H, Sun K, Tao W (2006). Optimization of the medium composition for production of mycelial biomass and exo-polymer by *Grifola frondosa* GF9801 using response surface methodology. Bioresour Technol.

[CR15] Del Castillo E (2007). Process optimization: a statistical approach.

[CR16] Djekrif-Dakhmouche S, Gheribi-Aoulmi Z, Meraihi Z, Bennamoun L (2006). Application of a statistical design to the optimization of culture medium for α-amylase production by *Aspergillus niger* ATCC 16404 grown on orange waste powder. J Food Eng.

[CR17] Dubey KV, Vakil BV (2010). Development and characterization of bacteriophage resistant probitoic cultures. Earth Quest.

[CR18] Dubey K, Vakil B (2012) Development and characterization of bacteriophage resistant bacterial probiotic cultures using classical and recent bioanalytical techniques. Dissertation, University of Mumbai, India

[CR19] FAO Joint (2007) WHO working group on drafting guidelines for the evaluation of probiotics in food. Guidelines for the evaluation of probiotics in food: report of a Joint FAO/WHO working group on drafting guidelines for the evaluation of probiotics in food, London, ON, Canada, April 30 and May 1, 2002. http://www.who.int/foodsafety/fs_management/en/probiotic_guidelines.pdf

[CR20] Kumar S, Katiyar N, Ingle P, Negi S (2011). Use of evolutionary operation (EVOP) factorial design technique to develop a bioprocess using grease waste as a substrate for lipase production. Bioresour Technol.

[CR21] Kumar M, Jain AK, Ghosh M, Ganguli A (2012). Statistical optimization of physical parameters for enhanced bacteriocin production by *L. casei*. Biotechnol Bioprocess Eng.

[CR22] Lynch D (2003) EVOP design of experiments. In: SAE 2013 world conference and exhibition. SAE Technical Paper 2003-01-1015. doi:10.4271/2003-01-1015

[CR23] Mandenius C, Brundin A (2008). Bioprocess optimization using design-of-experiments methodology. Biotechnol Prog.

[CR24] Meena G, Gupta S, Majumdar G, Banerjee R (2011). Growth characteristics modeling of *Bifidobacterium bifidum* using RSM and ANN. Braz Arch Biol Technol.

[CR25] Monroy MR, De La Torre M (1996). Effect of the dilution rate on the biomass yield of *Bacillus thuringiensis* and determination of its rate coefficients under steady-state conditions. Appl Microbiol Biotechnol.

[CR26] Naveena BJ, Altaf M, Bhadriah K, Reddy G (2005). Selection of medium components by Plackett–Burman design for production of L (+) lactic acid by Lactobacillus amylophilus GV6 in SSF using wheat bran. Bioresour Technol.

[CR27] Pandey KR, Shinde PS, Vakil BV (2015). Evaluation of molecular variations in probiotic *Bacillus coagulans* and its bacteriophage resistant mutants. Int J Curr Microbiol Appl Sci.

[CR28] Plackett RL, Burman JP (1946). The design of optimum multifatorial experiments. Biometrika.

[CR29] Raissi S, Farsani RE (2009). Statistical process optimization through multi-response surface methodology. World Acad Sci Eng Technol.

[CR30] Rajendran A, Thirugnanam M, Thangavelu V (2007). Statistical evaluation of medium components by Plackett–Burman experimental design and kinetic modeling of lipase production by Pseudomonas fluorescens. Indian J Biotechnol.

[CR31] Rao N, Kumar S, Sharghi-Namini S, Ge R (2012). ADAMTS5 functions as an anti-angiogenic and anti-tumorigenic protein independent of its proteoglycanase activity. Am J Pathol.

[CR32] Rivera D (1999) Growth kinetics of *Bacillus thuringiensis*. Doctoral dissertation, The University of Western Ontario, London

[CR33] Sengupta S, Jana M, Sengupta D, Naskar A (2006). A note on the estimation of microbial glycosidase activities by dinitrosalicylic acid reagent. Appl Microbiol Biotechnol.

[CR34] Shuler ML, Kargi F (2002). Bioprocess engineering.

[CR35] Song C, Liu Z, Huang S, Zheng P, Yang P (2012). Probiotics promote endocytic allergen degradation in gut epithelial cells. Biochem Biophys Res Commun.

[CR36] Thirumavalavan K, Manikkandan TR, Dhanasekar R (2008). Batch fermentation kinetics of pullullan from *Aureobasidium pullulans* using low cost substrates. Biotechnology.

[CR37] Walters F, Parker L, Morgan S, Deming S (1991). Sequential Simplex optimization: a technique for improving quality and productivity in research, development, and manufacturing.

[CR38] Weiss RM, Ollis DF (1980). Extracellular microbial polysaccharides. I. Substrate, biomass, and product kinetic equations for batch xanthan gum fermentation. Biotechnol Bioeng.

[CR39] Zhang X, Zhou J, Fu W, Li Z, Zhong J, Yang J, Tan H (2010). Response surface methodology used for statistical optimization of jiean-peptide production by *Bacillus subtilis* cells adsorbed on wood chips. Electron J Biotechnol.

